# The autophagic marker p62 highlights Alzheimer type II astrocytes in metabolic/hepatic encephalopathy

**DOI:** 10.1111/neup.12660

**Published:** 2020-06-02

**Authors:** Ellen Gelpi, Jasmin Rahimi, Sigrid Klotz, Susanne Schmid, Gerda Ricken, Sara Forcen‐Vega, Herbert Budka, Gabor G. Kovacs

**Affiliations:** ^1^ Division of Neuropathology and Neurochemistry, Department of Neurology Medical University of Vienna Vienna Austria; ^2^ Neurological Tissue Bank of the Biobank of Hospital Clinic, August Pi i Sunyer Biomedical Research Institute (IDIBAPS) Barcelona Spain; ^3^ Department of Neurology and Karl Landsteiner Institute for Neuroimmunological and Neurodegenerative Disorders Danube Hospital Vienna Austria; ^4^ Neurology Department, Germans Trias i Pujol Hospital Autonomous University of Barcelona Barcelona Spain; ^5^ Center for Neurodegenerative Disease Research (CNDR), Institute on Aging and Department of Pathology and Laboratory Medicine University of Pennsylvania Philadelphia Pennsylvania USA; ^6^ Tanz Centre for Research in Neurodegenerative Disease (CRND) and Department of Laboratory Medicine and Pathobiology University of Toronto Toronto Ontario Canada; ^7^ Laboratory Medicine Program University Health Network Toronto Ontario Canada

**Keywords:** Alzheimer type II astrocytes, astrogliopathy, hepatic encephalopathy, metabolic encephalopathy, p62

## Abstract

Metabolic/hepatic encephalopathy is neuropathologically characterized by the presence of Alzheimer type II astrocytes (AA II) with large and clear nuclear morphology. To date, there is no good immunohistochemical marker to better identify these cells. Here, we assessed cases of hepatic encephalopathy of different etiologies by immunohistochemistry using an anti‐p62 antibody. We observed peripheral or diffuse nuclear staining of variable intensity in AA II in all cases but not in normal controls or reactive astrocytes. We conclude that p62 is a useful immunohistochemical marker for the identification of AA II and may be helpful for the neuropathological diagnosis of metabolic/hepatic encephalopathy in difficult or equivocal cases.

## INTRODUCTION

The presence of so‐called Alzheimer type II astrocytes (AA II) in the human brain usually reflects a metabolic disturbance caused by renal or, more frequently, hepatic dysfunction. Indeed, hepatic encephalopathy has been associated with the presence of AA II in the gray matter of different brain regions, usually involving the deep cortical layers, basal ganglia, and pontine nuclei.^1^ It is, therefore, considered to reflect a gliopathy.

**Figure 1:**
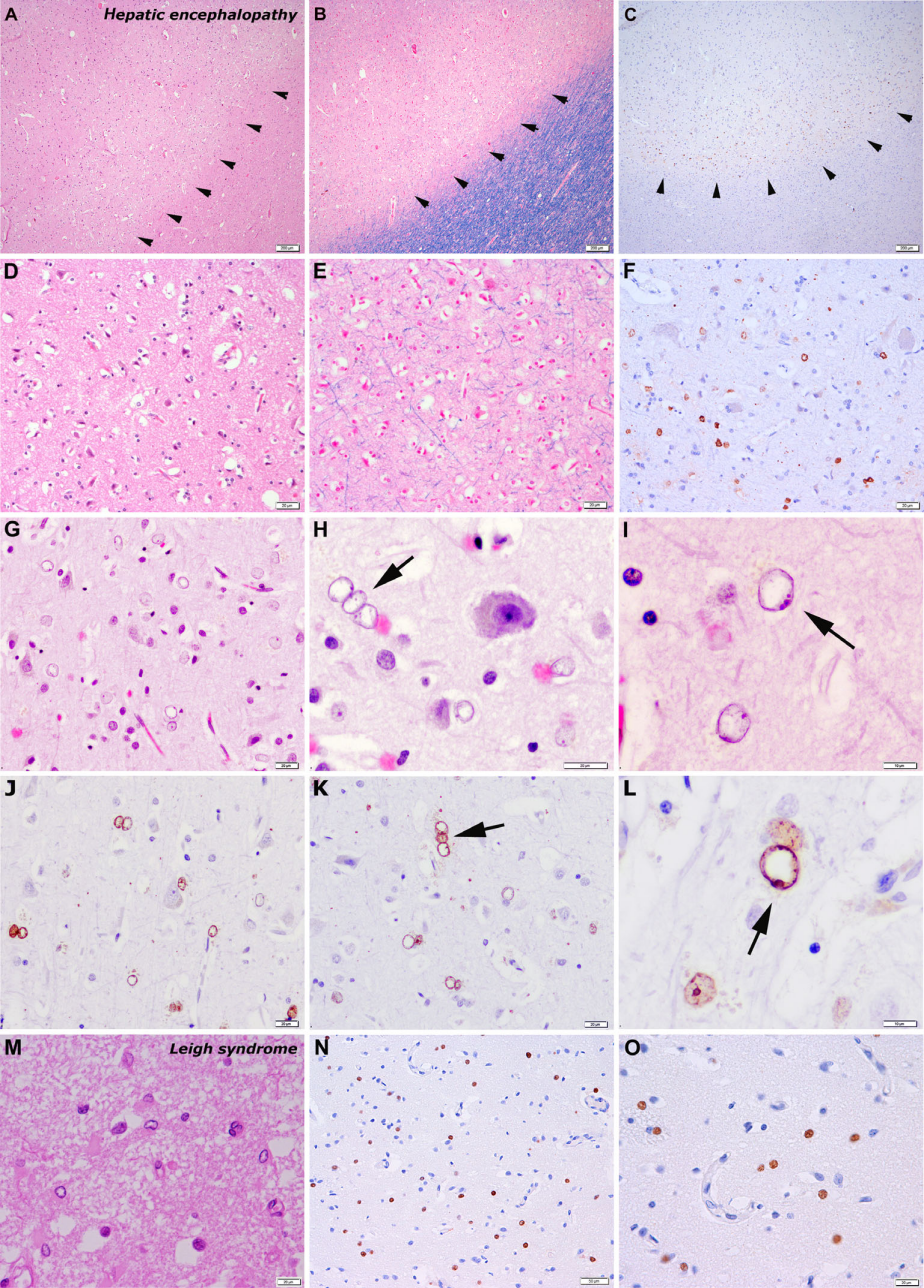
Microphotographs of brain sections of hepatic encephalopathy (A‐L) and mitochondrial encephalopathy/Leigh syndrome (M‐O). Laminar microvacuolation of the neuropil in cortical layers of the cerebrum is observed in a case of severe hepatic encephalopathy at a low magnification (arrows in A, B) and at a higher magnification (D, E). Abundant p62‐positive nuclei are identified in deep layers at a low magnification (arrows in C) and at a higher magnification (F). AA II have the characteristic enlarged cell nuclei and clear chromatin (G). AA II forming a triplet of nuclei (H). Small punctate condensations along the nuclear membrane in an AA II (I). p62 immunohistochemistry strongly labels the nuclei of AA II, including the peripheral nuclear membrane condensations (arrows in L). (M‐O) In a case of Leigh syndrome, glial nuclei in the basal ganglia are also enlarged but show more prominent cytoplasm on HE stained sections than typical AA II (M). p62 shows intense and diffuse labeling of enlarged glial nuclei (N, O). HE (A, D, E, G, H, M), LFB‐HE (B), p62 immunohistochemistry (C, F, J‐L, N, O). Scale bars: 200 μm (A–C), 50 μm (N), 20 μm (D–H, J, K–M), 10 μm (I).

AA II are characterized by a larger nucleus than in resting or reactive fibrillary or gemistocytic astrocytes, a clear chromatin (Fig. 1G), and scarce cellular processes. They are frequently forming pairs: doublets, or even triplets^1^ (Fig. 1H). However, AA II may be difficult to visualize on sections stained with hematoxylin and eosin (HE), showing a spectrum of nuclear changes, from slight enlargement and chromatin loosening to a completely clear or empty appearance of the nucleus with a well‐defined membrane rim and peripheral dot‐like condensation (Fig. 1I, arrow). There is currently no good marker to specifically identify AA II: they are characteristically not or are poorly stained by glial fibrillary acidic protein (GFAP) immunohistochemistry, while they can be depicted using anti‐S‐100 protein antibodies.^1^, ^2^ However, S‐100 protein is not a specific marker of astrocytes and labels most glioneuronal elements. Because the neuropathological diagnosis of metabolic encephalopathy may be difficult in its early or less severe disease stages, the application of a reliable immunohistochemical marker would be helpful to objectively support this diagnosis in the routine diagnostic or experimental settings.

Through staining of postmortem brains for other diagnostic purposes, we observed an intense staining of AA II nuclei using p62 immunohistochemistry in patients with metabolic encephalopathy. This observation prompted us to systematically assess glial p62 immunoreactivity in hepatic encephalopathy of different etiologies and to compare the staining pattern with other conditions characterized by prominent reactive gliosis.

## MATERIALS AND METHODS

Postmortem brains were selected from the archives of the Institute of Neurology of the Medical University of Vienna and the Neurological Tissue Bank of the IDIBAPS Biobank in Barcelona. The use of brain tissue for research was approved by the respective institutional ethics committees and conforms to the provisions of the Declaration of Helsinki.

Formalin‐fixed, paraffin‐embedded tissue blocks from the frontal, temporal and occipital cortices, anterior and posterior basal ganglia, thalamus, midbrain, pons and cerebellum were selected, and 5‐μm‐thick sections were stained with hematoxylin and eosin (HE) and Luxol fast blue (LFB) and HE (LFB‐HE), where the presence of AA II was assessed as present/absent (Table 1). One region per case with obvious AA II was first stained by immunohistochemistry using a commercial monoclonal anti‐p62 antibody (clone 3/p62 ligand, dilution 1:500; BD‐Transduction Laboratories, Franklin Lakes, NJ, USA). Then, selected cases of hepatic diseases (Table 2) were immunostained for p62 in the frontal cortex, basal ganglia, and pons, and in one case, a detailed mapping of p62 distribution was performed (Table 1). Antigen retrieval was performed by boiling the sections in citrate buffer at pH 6.0 for 20 min. The immunoreaction was visualized by the polymer‐immunocomplex method using an Envision System kit (Dako, Glostrup, Denmark), and 3,3'‐diaminobenzidine was used as chromogen. For double immunofluorescence labeling, the anti‐p62 antibody was combined with antibodies against S‐100 protein (rabbit polyclonal, dilution 1:2000; Dako), GFAP (rabbit polyclonal, dilution 1:5000; Dako), and tubulin polymerization‐promoting protein (TPPP/p25 (rabbit polyclonal, dilution 1:2000; non‐commercial). It has been shown that TPPP is mainly expressed in differentiated oligodendrocytes of the central nervous system (CNS),^3^ After blocking of autofluorescence with Sudan Black B, antibody binding immunoreactivities was visualized with secondary antibodies such as anti‐mouse IgG conjugated with Alexa Fluor488 (Thermo Fisher Scientific, Waltham, MA, USA) at a dilution of 1:800 and anti‐rabbit IgG conjugated with Cy3 (Thermo Fisher Scientific) at a dilution of 1:1000.

**Table 1:** Main features of cases with hepatic encephalopathy of different etiologies and anatomical distribution of suspected AA II

Order	Age	Gender	Cause of hepatic/renal damage	Metabolic encephalopathy severity HE	Neuropathological findings	Suspected Alzheimer II astrocytes on HE stained sections												
						Frontal	Cingulum	Parietal	Temporal	Occipital	Hippocampus	BBGG	Amygdala	Thalamus	Midbrain	Pons	Medulla obl	cbl + dentate
1	79	m	Hepatitis C and liver cirrhosis	Prominent	1) Metabolic encephalopathy; 2) Incidental LB pathology (Braak 2); 3) PART (Braak II) + mild CAA	**+**	**+**	**+**	**+**	**+**	**+**	**+**	**+**	**+**	**+**	**+**	**+**	**+**
2	77	m	Hepatitis C	Moderate	1) Metabolic encephalopathy; 2) Alzheimer's disease neuropathologic changes A3B3C2; 3) Incidental LB pathology olfactory bulb only	**+**	**+**	**+**	**+**	**−**	**+**	**+**	**−**	**+**	**−**	**+**	**+**	**+**
3	63	f	Hepatitis B, liver cirrhosis	Moderate	1) Metabolic encephalopathy; 2) Acute ischemic stroke	**+**	**+**	**+**	**+**	**+**	**+**	**+**	**+**	**+**	**+**	**+**	**+**	**+**
**4**	85	f	Alcohol abuse, chronic renal insufficiency	Moderate	1) Metabolic encephalopathy, 2) PART (Braak II) + mild CAA; 3) LATE	**3/+**	**3/+**	**2/+**	**3/+**	**1/+**	**0/s**	**2/+**	**0/s**	**1/+**	**2/+**	**0/+**	**0/−**	**0/+**
5	71	f	Alcohol abuse, liver cirrhosis, chronic renal insufficiency	Moderate	1) Metabolic encephalopathy; 2) Mild cerebellar atrophy; 3) PART (Braak II)	**+**	**−**	**−**	**−**	**−**	**+**	**+**	**+**	**+**	**+**	**+**	**+**	**+**
6	59	f	Alcohol abuse, liver cirrhosis	Moderate	1) Metabolic encephalopathy; 2) Acute Wernicke encephalopathy; 3) SVD	**+**	**+**	**+**	**+**	**+**	**+**	**+**	**+**	**+**	**+**	**+**	**+**	**+**
7	59	m	Alcohol abuse, liver cirrhosis, hepatocarcinoma	Prominent	1) Metabolic encephalopathy	**+**	**+**	**+**	**+**	**+**	**+**	**+**	**+**	**+**	**+**	**+**	**+**	**+**
8	65	m	Liver cirrhosis	Prominent	1) Metabolic encephalopathy; 2) Morel cortical laminar sclerosis; 3) Focal subarachnoid bleeding	**+**	**+**	**+**	**+**	**+**	**+**	**+**	**−**	**+**	**+**	**+**	**+**	**+**
9	70	f	Primary biliary cirrhosis	Prominent	1) Metabolic encephalopathy with focal spongy polio‐ encephalopathy	**+**	**+**	**+**	**+**	**+**	**+**	**+**	**+**	**+**	**+**	**+**	**+**	**+**
10	64	f	Autoimmune hepatitis, liver cirrhosis	Prominent	1) Metabolic encephalopathy; 2) Posthypoxic, postictal encephalopathy and bilateral hippocampal sclerosis	**+**	**+**	**+**	**+**	**+**	**+**	**+**	**+**	**+**	**+**	**+**	**+**	**+**
11	84	f	Hepatocellular carcinoma	Mild	1) Metabolic encephalopathy; 2) PART (Braak IV); 3) Incidental LB pathology (Braak 2)	**+**	**+**	**+**	**+**	**+**	**+**	**+**	**+**	**+**	**+**	**+**	**+**	**+**
12	58	m	Hepatocellular carcinoma	Prominent	1) Metabolic encephalopathy; 2) AgD Saito I	**+**	**+**	**+**	**+**	**+**	**+**	**+**	**+**	**+**	**+**	**+**	**+**	**+**
13	51	m	Metastatic unknown primary tumor with subtotal liver destruction	Prominent	1) Metabolic encephalopathy; 2) Pontine micro‐metastasis carcinoma	**+**	**+**	**+**	**+**	**+**	**+**	**+**	**+**	**+**	**+**	**+**	**+**	**+**
14	81	f	Metastatic pancreas carcinoma, liver necrosis	Moderate	1) Metabolic encephalopathy; 2) Mild ARP (Braak II, CERAD B)	**+**	**+**	**+**	**+**	**+**	**+**	**+**	**+**	**+**	**+**	**+**	**+**	**+**
15	85	m	Metastatic prostate carcinoma including liver	Mild	1) Metabolic encephalopathy; 2) Subdural hemorrhage; 3) Acute hypoxic‐ischemic neuronal damage in pons and cerebellum	**+**	**−**	**−**	**−**	**+**	**+**	**+**	**+**	**+**	**+**	**+**	**−**	**+**
16	78	f	Metastatic colon carcinoma including liver	Mild	1) Metabolic encephalopathy; 2) Mild ARP (Braak I, CERAD A) + mild CAA	**+**	**+**	**+**	**−**	**+**	**+**	**+**	**−**	**−**	**+**	**+**	**+**	**+**
17	58	f	B‐cell lymphoma diffuse, acute renal and hepatic failure	Moderate	1) Metabolic encephalopathy; 2) Lymphomatosis meningea	**+**	**+**	**+**	**+**	**+**	**+**	**+**	**+**	**+**	**+**	**+**	**+**	**+**
18	68	m	Sepsis	Moderate	1) Metabolic encephalopathy; 2) PART (Braak II)	**+**	**−**	**−**	**−**	**+**	**+**	**+**	**+**	**+**	**+**	**+**	**+**	**+**
19	52	m	Sepsis, cardiopulmonary reanimation	Moderate	1) Metabolic encephalopathy; 2) posthypoxic‐postischemic encephalopathy	**+**	**−**	**−**	**−**	**+**	**+**	**+**	**−**	**+**	**+**	**+**	**+**	**+**
20	15	f	Sepsis, vasculitis c‐ANCA	Moderate	1) Metabolic encephalopathy; 2) Multiple microbleeds	**+**	**+**	**+**	**+**	**+**	**+**	**+**	**+**	**+**	**+**	**+**	**+**	**+**
21	84	m	Sepsis, renal insufficiency	Mild	1) Metabolic encephalopathy; 2) SVD; 3) PART (Braak I)	**+**	**−**	**+**	**+**	**+**	**+**	**+**	**−**	**+**	**−**	**+**	**−**	**+**
22	0.5	f	Sepsis; pulmonary transplantation, fungal sepsis, pulmonary fibrosis, hepato‐splenomegalia and hepatic steatosis	Prominent	1) Metabolic encephalopathy; 2) Diffuse gliosis	**+**	**−**	**−**	**−**	**−**	**+**	**+**	**−**	**−**	**−**	**+**	**+**	**+**
23	69	m	Sepsis, cor pulmonale, hepatic steatosis	Mild	1) Metabolic encephalopathy ‐ Wernicke encephalopathy; 2) Incidental LB pathology (Braak 3); 3) PART (Braak II).	**+**	**+**	**+**	**+**	**+**	**+**	**+**	**+**	**+**	**+**	**+**	**+**	**+**
24	7	m	Sepsis, cardial transplantation, fungal pneumonia and sepsis, acute liver necrosis	Prominent	1) Metabolic encephalopathy; 2) Hypoxic‐ischemic damage with cortical necrosis; 3) Fungal microabscesses	**+**	**+**	**+**	**+**	**+**	**+**	**+**	**+**	**+**	**+**	**+**	**+**	**+**
25	64	m	Pericardial tamponade, congestive liver	Mild	1) Metabolic encephalopathy; 2) Mild acute hypoxic‐ischemic neuronal damage; 3) PART (Braak I)	**+**	**+**	**+**	**+**	**+**	**+**	**+**	**+**	**+**	**+**	**+**	**+**	**+**
26	69	f	Cardiac and hepatic fibrosis, anemia perniciosa, colitis ulcerosa	Mild	1) Metabolic encephalopathy; 2) Arteriolosclerosis and status cribrosus; 3) PART (Braak II)	**+**	**+**	**+**	**+**	**+**	**+**	**+**	**+**	**+**	**+**	**+**	**+**	**+**
27	81	f	Cardial insufficiency	Moderate	1) Metabolic encephalopathy; 2) PART (Braak III)	**+**	**+**	**+**	**+**	**+**	**+**	**+**	**+**	**+**	**+**	**+**	**+**	**+**
28	1	m	Cardial malformation with insufficiency and hepatosplenomegalia	Prominent	1) Metabolic or hypoxic? encephalopathy; 2) malformation: Dandy‐Walker like and agenesis of olfactorius	**+**	**−**	**+**	**−**	**+**	**+**	**+**	**−**	**+**	**+**	**+**	**+**	**+**
29	74	f	Suprarenal insufficiency, insulinoma, multiple complications	Mild	1) Metabolic encephalopathy with Wernicke‐like changes and Morel cortical laminar sclerosis; 2) Mild ARP (Braak I, CERAD B)	**+**	**+**	**+**	**+**	**+**	**+**	**+**	**−**	**+**	**+**	**+**	**−**	**+**
30	85	f	Hepatic insufficiency, unknown origin	Moderate	1) Metabolic encephalopathy; 2) PART (Braak II); 3) Old infarct	**+**	**−**	**+**	**−**	**+**	**+**	**+**	**+**	**+**	**+**	**+**	**+**	**+**
31	2	m	Mitochondrial encephalopathy ‐ Leigh syndrome; hepatomegaly	Moderate	1) Metabolic‐mitochondrial encephalopathy consistent with Leigh‐syndrome	**−**	**+**	**+**	**−**	**−**	**+**	**+**	**−**	**+**	**+**	**−**	**+**	**+**

In case 4, a detailed anatomical mapping of p62 immunoreactivity in relation to the presence of AA II was performed (0, negative; 1, sparse stained glial nuclei; 2, moderate density of stained glial nuclei; 3, high density of stained nuclei. s, single; +, present; −, absent; n.a, not available. ARP, Alzheimer's disease‐related pathology; CAA, amyloid angiopathy; LATE, limbic age‐related TDP43 encephalopathy; LB, Lewy body; PART, primary age‐related tauopathy; SVD, small vessel disease.

**Table 2:** p62 Immunoreactivity in the frontal cortex, basal ganglia, and pons in selected patients with different ages, etiologies, and formalin fixation times

Order	Age	Weeks in formalin	Hepatic pathology	Glial p62 nuclear immunoreactivity		
				Frontal cortex	Basal ganglia	Pons
1	79	1	Hepatitis C/cirrhosis	0/+	1/+	0/s
4	85	1	Alcohol abuse/cirrhosis	3/+	3/+	0/s
22	0,5	2	Hepatic steatosis	1/+	2/+	1/+
20	15	2	Sepsis, vasculitis	2/+	0/+	2/+
19	52	3	Sepsis	0/+	0/+	0/+
13	51	4	Metastasis	2/+	2/+	0/+
31	2	4	Mitochondrial disorder/Leigh syndrome	0/−	3/+	2/+
14	81	> 4	Metastasis	3/+	3/+	0/+
7	59	6	Alcohol abuse/cirrhosis/hepatocellular carcinoma	3/+	3/+	2/+
11	84	6	Hepatocellular carcinoma	1/+	0/+	0/+
17	58	14	Acute hepatic and renal failure, B cell lymphoma	0/+	0/+	0/+
28	1	> 25	Cardiac malformation, hepatosplenomegaly	3/+	0/+	0/+
21	84	n.a.	Sepsis	0/+	0/+	0/+

We selected 31 cases with hepatic encephalopathy of viral (Hepatitis C), neoplastic (hepatocarcinoma and liver metastasis), alcoholic (liver cirrhosis), systemic (sepsis), and mitochondrial (Leigh syndrome) origins (Table 1). For additional comparison of the immunostaining pattern, we assessed different pathologies, including subacute stage of cerebral infarction with prominent reactive gliosis, as well as different neurodegenerative diseases with variable degrees of chronic reactive gliosis, including Alzheimer's disease, corticobasal degeneration, progressive supranuclear palsy, Parkinson's disease, frontotemporal lobar degeneration with inclusions immunoreactive for transactivation response DNA‐binding protein 43 kDa (TDP‐43), and Creutzfeldt–Jakob disease (one case each), as well as one normal brain.

Details of cases with hepatic/metabolic encephalopathy are shown in Table 1.

## RESULTS

**Figure 2:**
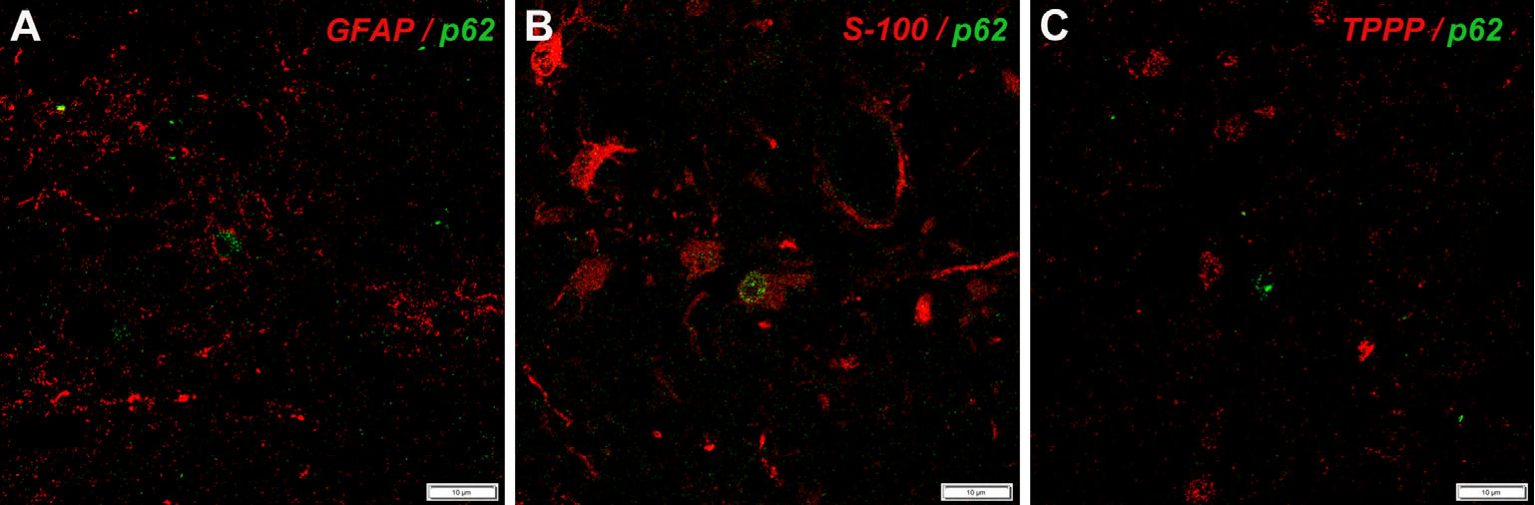
Microscopic findings of double immunofluorescence staining for p62 (green signal in A‐C) with GFAP (red signal in A), S‐100 protein (red signal in B)m and TPPP (red signal in C). p62 immunoreactivity is localized in some cells with delicate GFAP‐positive branching processes (A) and in diffusely stained S‐100 protein‐positive branching processes (B). In contrast, they do not coincide with TPPP‐positive oligodendrocytes (C).

Nuclear p62 staining was detected in enlarged glial cells of the gray matter that were consistent with AA II on HE‐stained sections (Fig. 1J–L). This immunopositivity was observed in all cases with hepatic encephalopathy of different etiologies, except for some of septic origin. Nuclear staining for p62 in AA II was particularly intense in a case of mitochondrial encephalopathy (Leigh syndrome) (Fig. 1M–O). Double immunofluorescence revealed p62‐positive nuclei in some delicate GFAP‐positive (Fig. 2A) and diffuse S‐100 protein‐positive cells (Fig. 2B) but not in TPPP/p25‐positive oligodendrocytes (Fig. 2C), thus supporting the astrocytic nature of the cells.

In cortical areas, AA II were best identified in deep layers. In severely affected cases, laminar microvacuolation of the neuropil in deep layers could be observed at a low magnification (arrows in Fig. 1A, B) and at a higher magnification (Fig. 1D, E). Here, abundant p62‐positive nuclei were identified at a low magnification (arrows in Fig. 1C) and at a higher magnification (Fig. 1F). When AA II showed the characteristic enlarged nuclei with clear chromatin, immunoreactivity was enhanced along the nuclear membrane and in the small punctate condensations (Fig. 1L). In cells with less obvious nuclear change, immunoreactivity was more diffuse.

The distribution and intensity of p62 immunoreactivity in AA II nuclei was not homogeneous among different brain areas of the same patient and between patients. The strongest signal was generally observed in cortical areas and was lower in the basal ganglia and pontine nuclei, but this was not uniform (Table 2). There were cases (e.g. case 4) showing a patchy distribution of immunoreactivity in the basal ganglia and cerebral cortex. In rare cases, cells that were considered to be AA II on HE‐stained sections were not or were only faintly immunoreactive for p62. Inversely, some cases with p62‐positive nuclei were not always clearly identifiable as AA II: for example, in the case of Leigh syndrome (Fig.1M–O) where immunopositivity filled the whole nucleus, in contrast to other cases with peripheral nuclear immunostaining. Bergmann's glia also showed relatively prominent p62 nuclear staining in one case of metabolic encephalopathy that had no prominent Purkinje cell loss (case 30). In contrast, other pathologies associated with Bergmann gliosis remained negative. Aquaporin 4 immunoreactivity was undetectable in AA II.

No immunostaining in glial nuclei was observed in the control and most neurodegenerative conditions with reactive astrogliosis, except for one case of corticobasal degeneration. In that case, p62‐positive nuclei corresponded to those of tau‐positive astrocytic plaques on adjacent tissue sections. Moreover, one case of sucacute stage cerebral infarction showed moderate nuclear immunoreactivity of large reactive astrocytes and also of some “eosinophilic neurons.” There were no differences in staining intensity that could be related to fixation time (Table 2) or postmortem delay (data not shown). No data on ammonia levels were available.

## DISCUSSION

We assessed the immunohistochemical expression of p62 in glial cells in different brain diseases and observed prominent nuclear staining of AA II in metabolic/hepatic encephalopathy, including astrocytes with no typical “clear” morphology. This was not observed in reactive astrocytes of most chronic neurodegenerative diseases, except for one case of corticobasal degeneration and subacute stage cerebral infarction, where nuclei of astrocytic plaques and large reactive astrocytes, respectively, were moderately labeled.

This observation suggests p62 as a very useful neuropathological marker of metabolic gliosis, particularly in hepatic encephalopathy.

Immunohistochemistry using anti‐p62 antibodies has proved very useful in the study of neurodegenerative diseases, as it is commonly found in neuronal cytoplasmic or nuclear inclusions (e.g. Alzheimer's disease, frontotemporal lobar degenerations, Lewy body diseases, or trinucleotide repeat disorders such as Huntington's disease). The presence of p62 is also a useful predictor of *C9orf72* expansion mutation when accumulated in granular neurons of the cerebellar cortex or hippocampal neurons.^4^, ^5^

p62 or sequestosome‐1 is a protein encoded by *SQSTM1* and is thought to target protein aggregates for lysosomal degradation, by binding to ubiquitinated proteins, among other functions.^6^, ^7^, ^8^ It is, therefore, considered to be an indicator of autophagic degradative activity. p62 itself is also degraded by autophagy. When autophagy is induced, it remains at low levels in the cell, while it accumulates when autophagy is deficient. It is also involved in protein aggregation, as shown for several proteinopathies associated with neurodegenerative conditions.^9^

Hepatic/metabolic encephalopathy has been reported to underlie several complex metabolic alterations,^10^ including mitochondrial dysfunction in astrocytes due to increased ammonia levels in blood and in the brain,^11^, ^12^ among others. Moreover, experimental studies have shown an involvement of mitophagy and autophagy^13^ in the pathogenesis of hepatic encephalopathy. In particular, treatment of cultured rat astrocytes with low concentrations of NH_4_Cl induced autophagy, while with higher concentrations from 2 mM onwards, NH_4_Cl inhibited autophagy in astrocytes in a time‐and dose‐dependent manner.^12^ These findings may provide one explanation for why high ammonia levels can induce the accumulation of p62 through inhibition of autophagy. In addition, exposure of astrocytes to ammonia also induces astrocytic swelling, which can be exacerbated by cytokines/inflammatory mediators.^14^ Some experimental studies have also shown that increased plasma membrane aquaporin 4 levels contribute to the astrocytic swelling/brain edema in hepatic encephalopathy.^10^ We found no increased immunoreactivity for aquaporin 4 in AA II. However, the detailed mechanism of peripheral and diffuse nuclear staining for p62 in hepatic/metabolic encephalopathy remains to be elucidated.

We observed a somewhat uneven distribution of p62‐positive AA II within the same brain area and between different brain areas and cases. It could be postulated that this might be related to levels of ammonia and/or duration or even to the cause of hepatic damage, reflecting an evolutive process of metabolic alterations of astrocytes. While it is generally considered that ammonia levels are positively related to the severity of hepatic encephalopathy, they are not always determinant as other factors may exacerbate it,^10^, ^15^ and they do not necessarily influence patient management.^16^ Unfortunately, we do not have enough data on ammonia levels or details on the duration of hepatic disease. Moreover, there was no particular difference in staining intensity depending on the etiology of liver damage, and it was apparently also not influenced by postmortem delay or formalin fixation time.

In summary, the postmortem neuropathological diagnosis of metabolic/hepatic encephalopathy has been a somewhat subjective, not always unequivocal diagnosis and can represent a challenge, particularly in less obvious stages. Even if not absolutely specific, we consider p62 as a useful immunohistochemical marker to visualize AA II. It can improve and objectify the identification of metabolic encephalopathy/gliopathy in postmortem brain tissue, in supplementation of classical HE staining features. Why p62 accumulates in the nucleus is, however, still unclear and deserves further investigation, particularly to better understand metabolic disturbances of astrocytes and their relationship with autophagy.

## DISCLOSURE

The authors received no financial support for this work and have no relationships that may pose a conflict of interest.

## References

[1] Kril J , Chimelli L , Morris CM , Harris JB . Nutritional and toxic diseases In: Love S, Budka H, Ironside JW, Perry A, (eds). Greenfield's Neuropathology, 9th edn, vol. 1 Chapter 9. Boca Raton, FL: CRC Press, 2015; 596.

[2] Kimura T , Budka H . Glial fibrillary acidic protein (GFAP) and S‐100 protein in human hepatic encephalopathy: immunocytochemical demonstration of dissociation of two glia‐associated proteins. Acta Neuropathol 1986; 70: 17–21. 3727931 10.1007/BF00689509

[3] Lehotzky A , Lau P , Tokési N , Muja N , Hudson LD , Ovádi J . Tubulin polymerization‐promoting protein (TPPP/p25) is critical for oligodendrocyte differentiation. Glia 2010; 58: 157–168. 19606501 10.1002/glia.20909 PMC2785070

[4] Al‐Sarraj S , King A , Troakes C *et al* p62 positive, TDP‐43 negative, neuronal cytoplasmic and intranuclear inclusions in the cerebellum and hippocampus define the pathology of C9orf72‐linked FTLD and MND/ALS. Acta Neuropathol 2011; 122: 691–702. 22101323 10.1007/s00401-011-0911-2

[5] Ramos‐Campoy O , Ávila‐Polo R , Grau‐Rivera O *et al* Systematic screening of ubiquitin/p62 aggregates in cerebellar cortex expands the neuropathological phenotype of the C9orf72 expansion mutation. J Neuropathol Exp Neurol 2018; 77: 703–709. 29889265 10.1093/jnen/nly047

[6] Cuyvers E , van der Zee J , Bettens K *et al* Genetic variability in SQSTM1 and risk of early‐onset Alzheimer dementia: a European early‐onset dementia consortium study. Neurobiol Aging 2015; 36: 2005.e15–2005.e22. 10.1016/j.neurobiolaging.2015.02.014 25796131

[7] Seibenhener ML , Babu JR , Geetha T , Wong HC , Krishna NR , Wooten MW . Sequestosome 1/p62 is a polyubiquitin chain binding protein involved in ubiquitin proteasome degradation. Mol Cell Biol 2004; 24: 8055–8068. 15340068 10.1128/MCB.24.18.8055-8068.2004 PMC515032

[8] Bjørkøy G , Lamark T , Johansen T . p62/SQSTM1: a missing link between protein aggregates and the autophagy machinery. Autophagy 2006; 2: 138–139. 16874037 10.4161/auto.2.2.2405

[9] Rué L , López‐Soop G , Gelpi E , Martínez‐Vicente M , Alberch J , Pérez‐Navarro E . Brain region‐ and age‐dependent dysregulation of p62 and NBR1 in a mouse model of Huntington's disease. Neurobiol Dis 2013; 52: 219–228. 23295856 10.1016/j.nbd.2012.12.008

[10] Jayakumar AR , Norenberg MD . Hyperammonemia in hepatic encephalopathy. J Clin Exp Hepatol 2018; 8: 272–280. 30302044 10.1016/j.jceh.2018.06.007 PMC6175739

[11] Bai Y , Wang Y , Yang Y . Hepatic encephalopathy changes mitochondrial dynamics and autophagy in the substantia nigra. Metab Brain Dis 2018; 33: 1669–1678. 29998403 10.1007/s11011-018-0275-6

[12] Lu KH . Involvement of autophagy and mitophagy in the pathogenesis of hepatic encephalopathy (Doctoral Thesis). 2018 Available from URL: https://docserv.uni‐duesseldorf.de/servlets/DerivateServlet/Derivate‐49390/Lu%20Kaihui%20Thesis%202018%20pdfa‐b1.pdf (Accessed January 30, 2020)

[13] Soria LR , Brunetti‐Pierri N . Ammonia and autophagy: an emerging relationship with implications for disorders with hyperammonemia. J Inherit Metab Dis 2019; 42: 1097–1104. 30671986 10.1002/jimd.12061

[14] Rama Rao KV , Jayakumar AR , Tong X , Alvarez VM , Norenberg MD . Marked potentiation of cell swelling by cytokines in ammonia‐ sensitized cultured astrocytes. J Neuroinflammation 2010; 7: 66. 20942959 10.1186/1742-2094-7-66 PMC2964656

[15] Reuter B , Walter K , Bissonnette J *et al* Assessment of the spectrum of hepatic encephalopathy: a multi‐center study. Liver Transpl 2018; 24: 587–594. 29457869 10.1002/lt.25032 PMC5912984

[16] Haj M , Rockey DC . Ammonia levels do not guide clinical management of patients with hepatic encephalopathy caused by cirrhosis. Am J Gastroenterol 2019 (Oct 14). [Epub ahead of print]. 10.14309/ajg.0000000000000343 31658104

